# Quercetin Inhibits Inflammatory Response Induced by LPS* from Porphyromonas gingivalis* in Human Gingival Fibroblasts via Suppressing NF-*κ*B Signaling Pathway

**DOI:** 10.1155/2019/6282635

**Published:** 2019-08-20

**Authors:** Gang Xiong, Wansheng Ji, Fei Wang, Fengxiang Zhang, Peng Xue, Min Cheng, Yanshun Sun, Xia Wang, Tianliang Zhang

**Affiliations:** ^1^Department of Condition Assurance, Affiliated Hospital of Weifang Medical University, Weifang Medical University, Weifang 261031, China; ^2^Department of Gastroenterology, Affiliated Hospital of Weifang Medical University, Weifang Medical University, Weifang 261031, China; ^3^School of Public Health and Management, Weifang Medical University, Weifang 261053, China; ^4^Collaborative Innovation Center of Prediction and Governance of Major Social Risks in Shandong, Weifang Medical University, Weifang 261053, China; ^5^Weifang Key Laboratory for Food Nutrition and Safety, Weifang Medical University, Weifang 261053, China; ^6^School of Clinical Medicine, Weifang Medical University, Weifang, 261053, China; ^7^Experimental Center for Medical Research, Weifang Medical University, Weifang 261053, China

## Abstract

Quercetin, a natural flavonol existing in many food resources, has been reported to be an effective antimicrobial and anti-inflammatory agent for restricting the inflammation in periodontitis. In this study, we aimed to investigate the anti-inflammatory effects of quercetin on* Porphyromonas gingivalis* (*P. gingivalis*) lipopolysaccharide- (LPS-) stimulated human gingival fibroblasts (HGFs). HGFs were pretreated with quercetin prior to LPS stimulation. Cell viability was evaluated by 3-[4,5-dimethylthiazol-2-yl]-2,5-diphenyltetrazolium bromide (MTT) assay. The levels of inflammatory cytokines, including interleukin-1*β* (IL-1*β*), interleukin-6 (IL-6), and tumor necrosis factor-*α* (TNF-*α*), along with chemokine interleukin-8 (IL-8), were determined by enzyme-linked immunosorbent assay (ELISA). The mRNA levels of IL-1*β*, IL-6, IL-8, TNF-*α*, I*κ*B*α*, p65 subunit of nuclear factor-kappa B (NF-*κ*B), peroxisome proliferator-activated receptor-*γ* (PPAR-*γ*), liver X receptor *α* (LXR*α*), and Toll-like receptor 4 (TLR4) were measured by real-time quantitative PCR (RT-qPCR). The protein levels of I*κ*B*α*, p-I*κ*B*α*, p65, p-p65, PPAR-*γ*, LXR*α*, and TLR4 were characterized by Western blotting. Our results demonstrated that quercetin inhibited the LPS-induced production of IL-1*β*, IL-6, IL-8, and TNF-*α* in a dose-dependent manner. It also suppressed LPS-induced NF-*κ*B activation mediated by TLR4. Moreover, the anti-inflammatory effects of quercetin were reversed by the PPAR-*γ* antagonist of GW9662. In conclusion, these results suggested that quercetin attenuated the production of IL-1*β*, IL-6, IL-8, and TNF-*α* in* P. gingivalis *LPS-treated HGFs by activating PPAR-*γ* which subsequently suppressed the activation of NF-*κ*B.

## 1. Introduction

More than 50% of the adult worldwide are affected by periodontal disease which can lead to the destruction of tooth-supporting tissues and even teeth loss [1]. Dental plaque (suggested initiative factor for periodontitis) and host immune response contribute to the pathogenesis of periodontitis [2]. Destruction of periodontal tissues is considered mainly due to an inappropriate host response to dental plaque or the corresponding microbial products such as lipopolysaccharide (LPS). Inflammatory mediators were found elevated accordingly in subjects with severe periodontitis [3].* Porphyromonas gingivalis *(*P. gingivalis*) has been regarded as one of the most important pathogens causing periodontal disease [4]. It leads to the destruction of teeth supporting tissues by inducing host and immune response [5]. LPS from* P. gingivalis* is considered to be a major virulence factor for periodontal inflammation [6].

Human gingival fibroblasts (HGFs), predominating in gingival connective tissue, are closely related to remodeling of periodontal soft tissues [7]. They can recognize LPS and mediate host immune response in periodontal lesions through interacting with bacteria directly [8]. They have been suggested a vital role in regulating the production of many inflammatory mediators such as interleukin-1*β* (IL-1*β*), interleukin-6 (IL-6), interleukin-8 (IL-8), and tumor necrosis factor-*α* (TNF-*α*) within gingival tissue [9]. These mediators are found upregulated in both gingival crevicular fluid (GCF) and periodontal tissues of periodontal patients [10]. Therefore, suppressing these inflammatory mediators or blocking the involved signaling pathway may help to restrict the initiation and progression of periodontal disease [11].

As one of the best characterized Toll-like receptors (TLRs) and principal receptor of LPS, TLR4 which can be generated in HGFs is involved in the production of inflammatory mediators and activation of downstream transcription factors such as nuclear factor-kappa B (NF-*κ*B) induced by LPS [12]. The upregulated TLR4 and inflammatory mediators aforementioned contribute to the injury of periodontal tissues [13]. Role of both peroxisome proliferator-activated receptor-*γ* (PPAR-*γ*) and liver X receptor *α* (LXR*α*) in regulating immune response has been demonstrated previously, and LPS-induced inflammatory responses can be inhibited by the activation of either PPAR-*γ* or LXR*α* [14, 15].

There exists a strong positive correlation between periodontal disease and many systemic ones such as cardiopathy [16]. Hence, preventing periodontal disease is beneficial to the health of whole body. Although conventional procedures such as brushing and flossing are generally effective to get rid of dental plaque and manage the progression of periodontal disease, the derived damage such as gingival recession and cementum abrasion has also been reported [17]. In addition, long-term use of antibiotics such as tetracycline and amoxicillin may result in increased drug-resistant resident microbial strains [18]. Since inflammatory responses can be suppressed by reducing inflammatory mediators, bioactive products capable to inhibit these mediators may be helpful to treating periodontal disease [11]. Quercetin (Q), a natural flavonol existing in many food resources such as vegetables and fruits, exhibited long-lasting and strong anti-inflammatory capability in various types of cells in both animal and human models [19]. It suppressed the production of cyclooxygenase (COX) and lipoxygenase (LOX) induced typically by inflammation in vitro [20]. Its anti-inflammatory activity was also reported in vivo [21]. It has been reported to be an effective antimicrobial and anti-inflammatory agent for restricting the inflammation in periodontitis [22, 23]. To the best of our knowledge, the anti-inflammatory effects of quercetin on LPS-stimulated HGFs have not been reported. In the present work, we aimed to investigate the anti-inflammatory effects of quercetin on* P. gingivalis* LPS-stimulated HGFs and the underlying anti-inflammatory mechanism.

## 2. Materials and Methods

### 2.1. Chemicals and Reagents

Quercetin was bought from the National Institute for Pharmaceutical and Biological Products Control (Beijing, China). 3-[4,5-dimethylthiazol-2-yl]-2,5-diphenyltetrazolium bromide (MTT) and bicinchoninic acid (BCA) protein concentration determination kits were purchased from Solarbio Life Science Co., Ltd (Beijing, China). LPS from* P. gingivalis *was supplied by Invitrogen (St. Louis, CA, USA). Rabbit monoclonal antibodies against I*κ*B*α*, phosphorylated I*κ*B*α* (p-I*κ*B*α*), p65 subunit of nuclear factor-kappa B (NF-*κ*B) (p65), phosphorylated p65 (p-p65) and *β*-actin were purchased from Cell Signaling Technology Inc (Beverly, MA, USA), with rabbit monoclonal antibodies against PPAR-*γ*, LXR*α* and TLR4 from Abcam Inc. (Cambridge, UK). Horseradish peroxidase (HRP)-conjugated goat anti-rabbit IgG was provided by Biosynthesis Biotechnolgogy Co., Ltd (Beijing,China). Enhanced chemiluminescence (ECL) kit was bought from Yanxi Co., Ltd (Shanghai, China). And the enzyme-linked immunosorbent assay (ELISA) kits were provided by Westtang Co., Ltd (Shanghai, China). All the other reagents used in the present work are of analytical grade.

### 2.2. Cell Culture

HGFs were purchased from Bena Culture Collection (BNCC, Jiangsu, China) and cultured in Dulbecco's Modified Eagle Medium (DMEM) containing 10% fatal bovine serum (FBS), penicillin (100 U/mL) and streptomycin (100 *μ*g/mL) at 37°C with 5% CO_2_. Cells between the 4th and 8th passages were used in the present work. Quercetin was dissolved in dimethyl sulfoxide (DMSO) and diluted using DMEM. This work was performed in five groups: control, LPS, LPS+5 *μ*M Q, LPS+10 *μ*M Q, and LPS+20 *μ*M Q. As to the last three groups employing quercetin and LPS, cells were pretreated with quercetin for 1 h prior to* P. gingivalis* LPS stimulation, with control and LPS groups pretreated using the same amount of DMSO instead.

### 2.3. Cell Viability

The effects of LPS and quercetin on cell viability of HGFs were measured by MTT assay based on the reduction of MTT to formazan by mitochondrial succinate dehydrogenase in viable cells. In brief, HGFs were seeded in a 96-well plate at a density of 1×10^4^ cells/well and treated with different concentrations of quercetin (final concentration at 5 *μ*M, 10 *μ*M and 20 *μ*M, respectively) with and without LPS (final concentration at 1 *μ*g/mL) for 24 h. Pre-treatment of quercetin was performed prior to the stimulation of LPS. Then 20 uL MTT (5 mg/mL) was added to each well, followed by incubation for another 4 h at 37°C with 5% CO_2_. After the medium was removed, 150 uL DMSO was added to each well to dissolve the insoluble formazan crystals in viable cells. Then the formazan concentrations were quantified by measuring the absorbance at 450 nm using a microplate spectraphotometer (Multiskan GO, Thermo, USA). Cell viability was expressed relative to the untreated control group which was regarded as 100%.

### 2.4. ELISA Assay

Protein levels of IL-1*β*, L-6, IL-8 and TNF-*α* in HGFs culture supernatants were determined using corresponding ELISA kits (Westtang Bio-tech, Shanghai, China) in accordance with the producer's instructions. Briefly, HGFs were seeded in a 24-well plate at a density of 2×10^5^ cells/ well. The cells were pretreated with quercetin for 1 h, followed by subsequent stimulation of LPS (final concentration at 1 *μ*g/mL) for 24 h. One hundred *μ*L standards or samples were added to each well of reaction plate and kept at 37°C for 40 min after being mixed fully. Then the reaction plate was washed 5 times with phosphate-buffered saline (PBS) and added 50 *μ*L biotinylated-antibodies working solution for each well and kept at 37°C for 20 min after being mixed fully. After being washed as aforementioned, 100 *μ*L enzyme conjugate working solution was added into each well and kept at 37°C for 10 min after being mixed fully. After being washed as aforementioned, 100 *μ*L TMB solution was added into each well and kept at 37°C for 15 min in dark prior to the addition of 100*μ*L stopping solution into each well. The absorbance at 450 nm was determined using a microplate spectraphotometer (Multiskan GO, Thermo, USA) within 30 min. The levels of IL-1*β*, L-6, IL-8 and TNF-*α* in cell culture supernatants, expressed as pg/mL, were quantified based on each corresponding standard curve. Samples were tested in triplicate and each experiment was repeated 3 times independently.

### 2.5. Real-Time Quantitative PCR (RT-qPCR)

mRNA levels of IL-1*β*, IL-6, IL-8, TNF-*α*, I*κ*B*α*, p65, PPAR-*γ*, LXR*α* and TLR4 in HGFs were evaluated by RT-qPCR. HGFs were seeded in a 6-well plate at a density of 1×10^6^ cells/well and pretreated with quercetin (final concentration at 5 *μ*M, 10 *μ*M and 20 *μ*M, respectively) for 1 h, followed by LPS stimulation for 3 h. Then the cells were washed three times using PBS buffer. Total RNA was extracted from HGFs using TRIzol reagent (Invitrogen, CA, USA) and the 1st strand cDNA was synthesized using a commercial kit (Takara, Otsu, Japan) according to the producer's instructions. Next, mRNA levels of the targets aforementioned were evaluated by RT-qPCR using an iQ5 RT-qPCR detection system (Bio-Rad, CA, USA) in a 20 *μ*L reaction system containing approximately 50 ng cDNA, 10 *μ*M of each primer and 10 *μ*L SsoFast EvaGreen Supermix (Bio-Rad Hercules, CA, USA). The RT-qPCR reaction starts with one cycle of 95°C for 30 s, followed by 40 cycles of 95°C for 5 s, 60°C for 30 s, and 72°C for 30 s. After RT-qPCR, a melting curve assay was immediately performed from 60°C to 95°C at a transition rate of 0.5°C/s to avoid the amplification of unspecific products. The primers used in the present work were presented in Table 1. The mRNA levels of these targets were calculated using the 2^−△△Ct^ method and normalized against *β*-actin which was used as an internal reference gene. The results were expressed as fold changes to control.

### 2.6. Western Blotting Analysis

Protein levels of I*κ*B*α*, p-I*κ*B*α*, p65, p-p65, PPAR-*γ*, LXR*α*, and TLR4 in HGFs were characterized by Western blotting. HGFs were seeded in a 6-well plate at a density of 1×10^6^ cells/well and pretreated with quercetin (final concentration at 5 *μ*M, 10 *μ*M, and 20 *μ*M, respectively) for 1 h, followed by LPS stimulation for 30 min. The cells were washed three times using cold PBS buffer before the total proteins were isolated using cold radio immunoprecipitation assay (RIPA, 150 mM NaCl, 50 mM Tris–HCl, pH 7.2, 1% Triton X-100, 0.1% SDS) (Solarbio, Beijing, China) lysis buffer supplemented with protease inhibitor of phenylmethylsulfonyl fluoride (PMSF) (final concentration at 1 mM) and phosphatase inhibitor cocktail (Cwbiotech, Jiangsu, China). Protein concentrations were measured using a BCA kit (Solarbio, Beijing, China) based on corresponding standard curves. Samples (30 *μ*g) were loaded to each well and separated on 10% sodium dodecyl sulfate-polyacrylamide gel electrophoresis (SDS-PAGE). Then the proteins were transferred onto polyvinylidene difluoride (PVDF) membrane (Solarbio, Beijing, China) at 300 mA under 4°C for 110 min. Under constant shaking, the membrane was blocked with 5% FBS (diluted in PBS) for 2 h and then incubated with corresponding rabbit primary monoclonal antibodies (diluted in 5% FBS) including I*κ*B*α* (1:500), p-I*κ*B*α* (1:500), p65 (1:500), p-p65 (1:500), *β*-actin (1:500), PPAR-*γ* (1:500), LXR*α* (1:1000) and TLR4 (1:200) at 4°C overnight. After being washed with cold PBS three times (5 min each), the membrane was incubated with HRP-conjugated secondary anti-rabbit antibodies diluted in 5% FBS (1:2000) for 2 h. After washing the membrane with cold PBS three times (5 min each), protein bands were visualized by ECL with a chemiluminescence gel imaging system (FluorChem Q, ProteinSimple, CA, USA). Band densities of proteins were analyzed using an ImageJ Gel Analysis tool (NIH, Bethesda, MD, United States) and normalized against *β*-actin which was used as an internal reference. The results were expressed as fold changes in relative densities to control.

### 2.7. Statistical Analyses

All experiments were performed three times independently and the results were presented as means ± SD. Data was analyzed by one-way ANOVA. Student–Newman–Keuls (SNK) post hoc test was employed to determine the significant difference among groups. All statistical analyses were carried out using SPSS software of version 19.0 (SPSS Inc., Chicago, IL, USA).* P *< 0.05 was considered statistically significant.

## 3. Results

### 3.1. Effects of Quercetin on Cell Viability of HGFs

**Figure 1:**
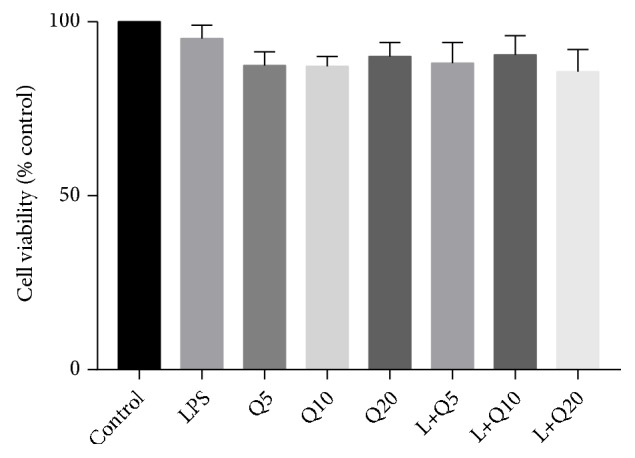
Effects of quercetin on cell viability of HGFs. Cells were cultured with quercetin at different final concentration of 5 *μ*M, 10 *μ*M, and 20 *μ*M, respectively, with and without LPS (final concentration at 1 *μ*g/mL) for 24 h. The values of three independent experiments are presented as means ± SD.

Effects of quercetin on cell viability of HGFs were evaluated by MTT assay. As is shown in Figure 1, quercetin at 5 *μ*M, 10 *μ*M, and 20 *μ*M, respectively, exerted no significant cytotoxic effects on cell viability of HGFs. Thus the effects of quercetin on LPS-stimulated HGFs in the present work are not attributed to the nonspecific cytotoxicity. Therefore, quercetin at these concentrations was used in the subsequent study.

### 3.2. Effects of Quercetin on Production of IL-1*β*, IL-6, IL-8, and TNF-*α* in LPS-Stimulated HGFs

**Figure 2:**
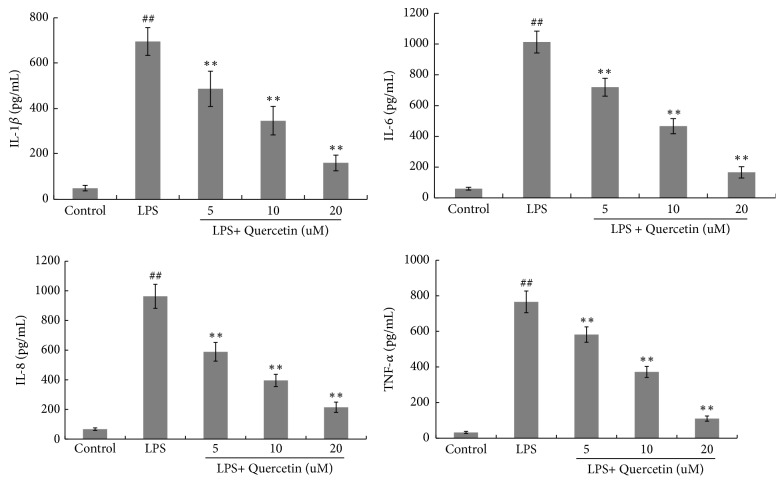
Quercetin inhibited production of IL-1*β*, IL-6, IL-8, and TNF-*α* in LPS-stimulated HGFs. HGFs were pretreated with quercetin at different final concentration of 5 *μ*M, 10 *μ*M and 20 *μ*M for 1 h, respectively, followed by LPS stimulation (final concentration at 1 *μ*g/mL) for 24 h. The values of three independent experiments are presented as means ± SD. ^##^* P*<0.01* vs.* control group; ^∗∗^* P*<0.01* versus* LPS group.

To investigate the anti-inflammatory effects of quercetin on LPS-stimulated HGFs, production of IL-1*β*, IL-6, IL-8, and TNF-*α* was detected by ELISA assay (Figure 2). Levels of these inflammatory mediators were significantly upregulated by LPS stimulation, compared with the control group. However, quercetin suppressed the LPS-induced production of IL-1*β*, IL-6, IL-8, and TNF-*α* in a dose-dependent manner (Figure 2).

### 3.3. Effects of Quercetin on mRNA Levels of IL-1*β*, IL-6, IL-8, TNF-*α*, I*κ*B*α*, p65, PPAR-*γ*, LXR*α*, and TLR4 in LPS-Stimulated HGFs

**Figure 3:**
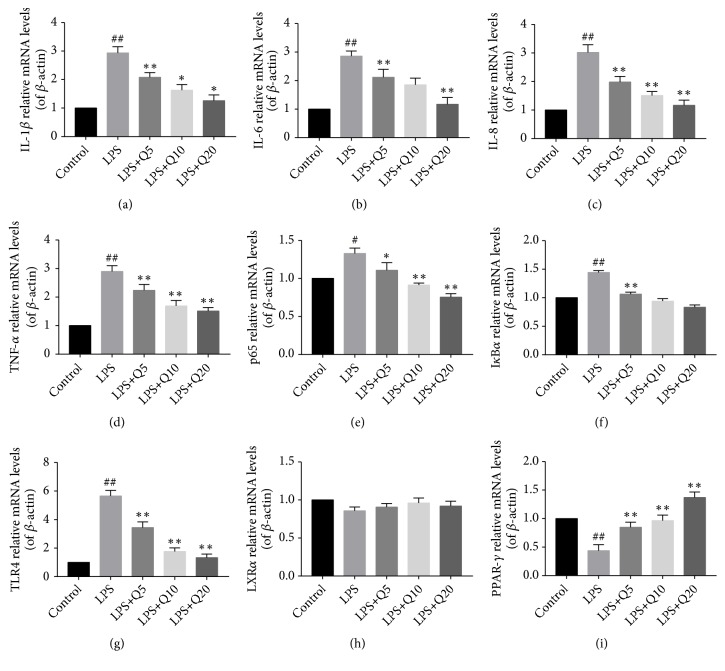
Effects of quercetin on mRNA levels of IL-1*β*, IL-6, IL-8, TNF-*α*, I*κ*B*α*, p65, PPAR-*γ*, LXR*α* and TLR4 in LPS-stimulated HGFs. HGFs were pretreated with quercetin at different final concentration of 5 *μ*M, 10 *μ*M, and 20 *μ*M for 1 h, respectively, followed by LPS stimulation (final concentration at 1 *μ*g/mL) for 3 h. A: IL-1*β*; B: IL-6; C: IL-8; D: TNF-*α*; E: p65; F: I*κ*B*α*; G: TLR4; H: LXR*α*; I: PPAR-*γ*. The values of three independent experiments are presented as means ± SD. ^#^*P*<0.05* versus* control group, ^##^*P*<0.01* versus* control group; *∗P*<0.05* versus* LPS group, ^∗∗^*P*<0.01* versus* LPS group.

mRNA levels of IL-1*β*, IL-6, IL-8, TNF-*α*, I*κ*B*α*, p65, PPAR-*γ*, LXR*α*, and TLR4 in LPS-stimulated HGFs were detected by RT-qPCR. As is shown in Figure 3, LPS stimulation significantly upregulated the mRNA levels of IL-1*β*, IL-6, IL-8, TNF-*α*, p65, I*κ*B*α*, and TLR4 but downregulated that of PPAR-*γ*, which could be suppressed by quercetin dose-dependently.

### 3.4. Effects of Quercetin on TLR4 Expression and NF-*κ*B Activation in LPS-Stimulated HGFs

**Figure 4:**
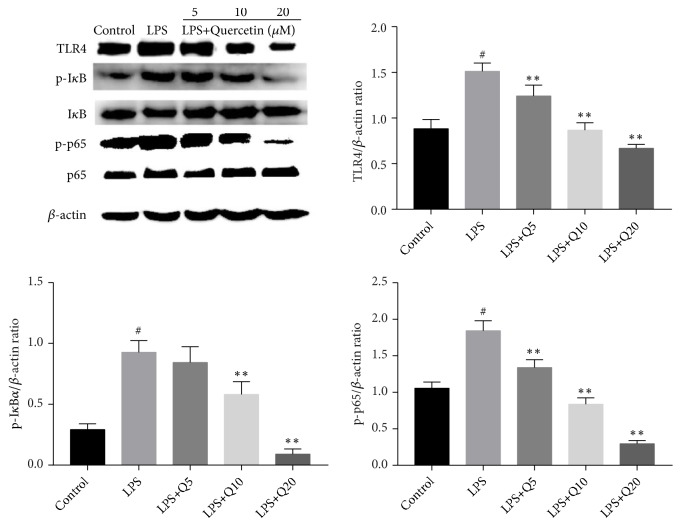
Effects of quercetin on TLR4 expression and NF-*κ*B activation in LPS-stimulated HGFs. HGFs were pretreated with quercetin at different final concentration of 5 *μ*M, 10 *μ*M, and 20 *μ*M for 1 h, respectively, followed by LPS stimulation (final concentration at 1 *μ*g/mL) for 30 min. The values of three independent experiments are presented as means ± SD. ^#^*P*<0.05* versus* control group; ^∗∗^*P*<0.01* versus* LPS group.

TLR4 serves as the main receptor of LPS. The import role of NF-*κ*B signaling pathway in regulating the production of inflammatory mediators has been reported previously. In the present work, we investigated the effects of quercetin on TLR4 expression and NF-*κ*B activation in LPS-stimulated HGFs. The results exhibited that LPS upregulated TLR4 expression and the phosphorylation of p65 and I*κ*B*α* significantly, which could be inhibited by quercetin in a dose-dependent manner, however (Figure 4).

### 3.5. Effects of Quercetin on Expression of PPAR-*γ* and LXR*α* in LPS-Stimulated HGFs

**Figure 5:**
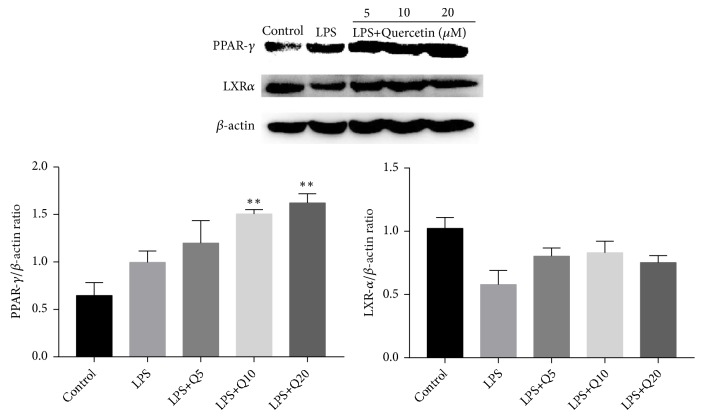
Effects of quercetin on expression of PPAR-*γ* and LXR*α* in LPS-stimulated HGFs. HGFs were pretreated with quercetin at different final concentration of 5 *μ*M, 10 *μ*M, and 20 *μ*M for 1 h, respectively, followed by LPS stimulation (final concentration at 1 *μ*g/mL) for 30 min. The values of three independent experiments are presented as means ± SD. ^∗∗^*P*<0.01* versus* LPS group.

Individual activation of PPAR-*γ* and LXR*α* has been suggested to exert anti-inflammatory effects. In the present work, we investigated the effects of quercetin on expression of PPAR-*γ* and LXR*α*. The results showed that the expression of PPAR-*γ* was upregulated significantly, with no significant change in that of LXR*α* (Figure 5). This suggests that the anti-inflammation effects of quercetin in LPS-stimulated HGFs are through the activation of PPAR-*γ*.

### 3.6. Anti-Inflammatory Effects of Quercetin on LPS-Stimulated HGFs Are PPAR-*γ*-Dependent

**Figure 6:**
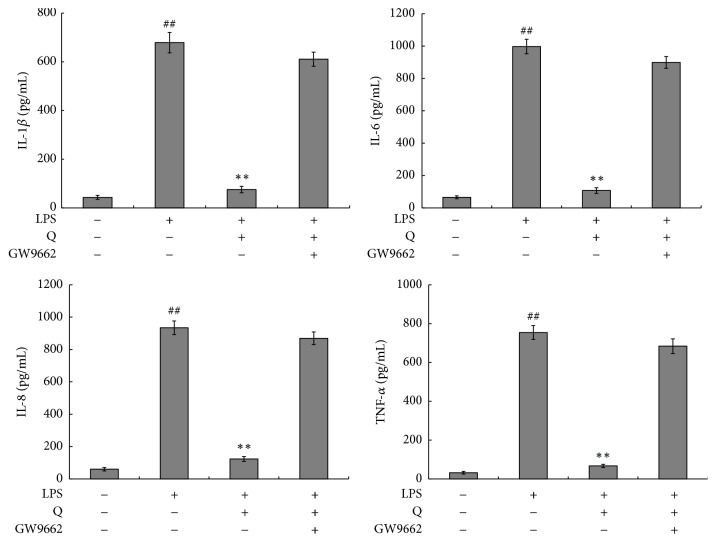
PPAR-*γ* antagonist of GW9662 reversed the anti-inflammatory effects of quercetin on LPS-stimulated HGFs. The values of three independent experiments are presented as means ± SD. ^##^*P*<0.01* versus* control group; ^∗∗^*P*<0.01* versus* LPS group.

Whether the anti-inflammatory effects of quercetin are PPAR-*γ*-dependent was investigated. The results showed that the inhibitory effects of quercetin on the production of IL-1*β*, IL-6, IL-8 and TNF-*α* were reversed by the PPAR-*γ* antagonist of GW9662 (Figure 6).

## 4. Discussion

It has been reported that inflammation plays an important part in the pathogenesis of periodontal disease [24]. Therefore, controlling inflammation is conducive to the treatment of periodontal disease [25]. Previous studies demonstrated the ability of many natural compounds to treat periodontal disease [26, 27]. Quercetin, a natural flavonol rich in fruits, vegetables and some berries, and so on, has exhibited good anti-inflammatory effects previously [20, 21]. In the present work, we investigated the anti-inflammatory effects of quercetin on LPS-stimulated HGF in vitro. The results showed that quercetin attenuated the production of inflammatory mediators of IL-1*β*, IL-6, IL-8 and TNF-*α* by suppressing NF-*κ*B signaling pathway.

The Gram-negative bacterium of* P. gingivalis* serves as the most important etiologic factor in periodontal disease [28]. As a crucial virulence factor for periodontitis,* P. gingivalis* LPS could induce the release of inflammatory mediators aforementioned in various cells such as HGFs, and result in a series of inflammatory reactions [29]. IL-1*β* can promote the production of IL-6 and its overproduction may initiate and facilitate the breakdown of connective tissue [30]. IL-6 contributes to the pathogenesis of periodontal diseases via inducing osteoclastogenesis, tissue destruction and bone resorption [31]. As a major chemoattractant, IL-8 can recruit neutrophils which can cause the destruction of normal periodontal tissues by releasing metalloproteinases [32]. Generated in early period of inflammation, TNF-*α* brings oxidative damage to periodontal tissues due to its most effectiveness in inducing superoxide production in HGFs [33]. Moreover, IL-1*β*, IL-8 and TNF-*α* exert dose- and time-dependent synergistic effects on the upregulation of IL-6, hundred times more potent than LPS dose [34]. It has been well documented regarding the association between these inflammatory mediators and the pathogenesis of periodontal disease [35]. And previous studies showed attenuated pathological process of periodontal disease by inhibiting these inflammatory mediators [36]. In the present study, the mRNA and protein levels of IL-1*β*, IL-6, IL-8 and TNF-*α* were significantly upregulated by the noncytotoxic concentration of LPS but down-regulated by noncytotoxic concentrations of quercetin, suggesting the anti-inflammatory capability of quercetin. And these inflammatory mediators can be down-regulated effectively by all the three concentrations of quercetin (5 *μ*M, 10 *μ*M, and 20 *μ*M, respectively). Similarly, pretreatment with quercetin at 20 *μ*M for 24 h could significantly attenuated trauma-induced TNF-*α* increase in H9c2 cells and pretreatment with quercetin at higher than 20 *μ*M exerted cytotoxicity [37]. However, quercetin was also reported to exert anti-inflammatory effects at dosage of as high as 100 *μ*M in pulmonary epithelial (A549) and N9 microglial cells [38, 39].

The vital role of NF-*κ*B signaling pathway in regulating inflammatory responses has been presented previously [40]. Periodontal inflammation may be relieved via the inhibition of NF-*κ*B activation [25]. We investigated the effects of quercetin on NF-*κ*B activation to study its anti-inflammation mechanism. The results showed that quercetin dose-dependently inhibited the LPS-induced NF-*κ*B activation and elevated TLR4 level. It has been reported that TLR4 could mediate the activation of NF-*κ*B in LPS-stimulated HGFs [41], which is consistent with the present work. TLR4 can induce the activation of NF-*κ*B and mitogen-activated protein kinases (MAPK) signaling pathways, both of which can predominately modulate the expression of inflammatory cytokines [40, 42]. In this study, I*κ*B*α* was used as an indicator of the status of NF-*κ*B activation [43]. Normally, NF-*κ*B is bound to its inhibitors (I*κ*B family) and locates in cytoplasm in an inactive form [44]. Under the stimulation of LPS, I*κ*B proteins are phosphorylated and degraded (Figure 4), and NF-*κ*B p65 may translocate from cytoplasm into the nucleus to upregulate the inflammatory mediators [45]. The present work demonstrated that quercetin suppressed LPS-induced NF-*κ*B activation and I*κ*B*α* phosphorylation in a dose-dependent manner (Figure 4). Similar anti-inflammatory effects of quercetin via inhibiting NF-*κ*B activation were also reported in other cell types [46, 47]. As to the mechanism of natural bioactive products against inflammation induced by LPS, some researchers support the direct inhibition of p65 nuclear translocation [48], while others claim the suppression of I*κ*Ba phosphorylation and degradation [49]. Whether the translocation of NF-*κ*B p65 can also be directly inhibited by quercetin in LPS-stimulated HGFs needs further verification. It is reported that quercetin could down-regulate the expression of inflammatory mediators through suppressing other signaling pathways such as MAPK [50]. Therefore, whether any other signaling pathway plays a role in the anti-inflammatory effects of quercetin in LPS-stimulated HGFs also needs further clarification.

PPAR-*γ* is a nuclear receptor and capable of regulating bone metabolism and inflammation [51]. The progression of experimental periodontitis in rats was attenuated significantly by the PPAR-*γ* agonist of rosiglitazone in a previous study [52]. Moreover, PPAR-*γ* could be activated by many natural products [53–55]. LXR*α*, a member of nuclear hormone superfamily, has been reported to be an important anti-inflammatory transcription factor involved in the development of inflammatory disease [56]. Agonists of LXR*α* have been shown to exert anti-inflammatory activity [57]. In addition, the activation of PPAR-*γ* or LXR*α* has been demonstrated to attenuate the inflammatory responses induced by LPS via suppressing the activation of NF-*κ*B [58, 59]. To clarify the anti-inflammatory mechanism of quercetin, its effects on the expression of PPAR-*γ* and LXR*α* were characterized. The results showed that quercetin significantly upregulated the expression of PPAR-*γ*, while exerting no significant effects on that of LXR*α*. In addition, the inhibitory effects of quercetin on the production of IL-1*β*, IL-6, IL-8 and TNF-*α* can be reversed by GW9662 which is a PPAR-*γ* antagonist. This is consistent with a previous study exploring the anti-inflammatory effects of asiatic acid on LPS-stimulated HGFs [60]. Therefore, the results suggest that quercetin attenuates LPS-induced inflammatory response in HGFs by activating PPAR-*γ*, which subsequently suppressed the activation of NF-*κ*B. Indeed, more blocking assays are needed in our future work to confirm whether quercetin pretreatment could inhibit the nuclear translocation of NF-*κ*B p65 by immunofluorescence staining and the activation of NF-*κ*B using a luciferase reporter gene assay. Moreover, a NF-*κ*B agonist can be used to confirm whether it could reverse the anti-inflammatory effects of quercetin on LPS-stimulated HGFs.

## 5. Conclusion

In conclusion, our results demonstrated that quercetin attenuated the production of inflammatory mediators of IL-1*β*, IL-6, IL-8, and TNF-*α* in* P. gingivalis *LPS-treated HGFs. The anti-inflammatory mechanism was through activating PPAR-*γ*, which subsequently suppressed the activation of NF-*κ*B induced by LPS. More in vivo studies are needed to evaluate the anti-inflammatory property of quercetin. The present work suggested a promising therapeutic potential for quercetin in treating periodontal disease.

## Figures and Tables

**Table 1:** Primers used for RT-qPCR.

Target	Primer Sequence (5'–3')
IL-1*β*	F: TAGGGCTGGCAGAAAGGGAACA
	R: GTGGGAGCGAATGACAGAGGGT

IL-6	F: CGCCTTCGGTCCAGTTGCC
	R: GCCAGTGCCTCTTTGCTGCTTT

IL-8	F: CTCTTGGCAGCCTTCCTGATTTC
	R: TTTTCCTTGGGGTCCAGACAGAG

TNF-*α*	F: AACATCCAACCTTCCCAAACGC
	R:TGGTCTCCAGATTCCAGATGTCAGG

I*κ*B*α*	F:CACTCCATCCTGAAGGCTACCAACTAC
	R: ATCAGCACCCAAGGACACCAAA

p65	F: AATGCTGTGCGGCTCTGCTTC
	R:CCGTGAAATACACCTCAATGTCCTCT

PPAR-*γ*	F: CGCCCAGGTTTGCTGAATGTG
	R: AGGGAAATGTTGGCAGTGGCTC

LXR*α*	F: TGATGTTCCCACGGATGCTAATG
	R: TTTGCCCTTCTCAGTCTGTTCCAC

TLR4	F: CACAGACTTGCGGGTTCTACATC
	R:GGACTTCTAAACCAGCCAGACCTTG

*β*-actin	F:GACTTAGTTGCGTTACACCCTTTCTTG
	R:CTGTCACCTTCACCGTTCCAGTTTT

## Data Availability

The data used to support the findings of this study are included within the article.
